# Functional complementation between transcriptional methylation regulation and post-transcriptional microRNA regulation in the human genome

**DOI:** 10.1186/1471-2164-12-S5-S15

**Published:** 2011-12-23

**Authors:** Zhixi Su, Junfeng Xia, Zhongming Zhao

**Affiliations:** 1 Department of Biomedical Informatics, Vanderbilt University School of Medicine, Nashville, TN 37232, USA; 2 MOE Key Laboratory of Contemporary Anthropology and Center for Evolutionary Biology, School of Life Sciences, Fudan University, Shanghai 200433, China; 3 Department of Psychiatry, Vanderbilt University School of Medicine, Nashville, TN 37232, USA; 4 Department of Cancer Biology, Vanderbilt University School of Medicine, Nashville, TN 37232, USA

## Abstract

**Background:**

DNA methylation in the 5' promoter regions of genes and microRNA (miRNA) regulation at the 3' untranslated regions (UTRs) are two major epigenetic regulation mechanisms in most eukaryotes. Both DNA methylation and miRNA regulation can suppress gene expression and their corresponding protein product; thus, they play critical roles in cellular processes. Although there have been numerous investigations of gene regulation by methylation changes and miRNAs, there is no systematic genome-wide examination of their coordinated effects in any organism.

**Results:**

In this study, we investigated the relationship between promoter methylation at the transcription level and miRNA regulation at the post-transcription level by taking advantage of recently released human methylome data and high quality miRNA and other gene annotation data. We found methylation level in the promoter regions and expression level was negatively correlated. Then, we showed that miRNAs tended to target the genes with a low DNA methylation level in their promoter regions. We further demonstrated that this observed pattern was not attributed to the gene expression level, expression broadness, or the number of transcription factor binding sites. Interestingly, we found miRNA target sites were significantly enriched in the genes located in differentially methylated regions or partially methylated domains. Finally, we explored the features of DNA methylation and miRNA regulation in cancer genes and found cancer genes tended to have low methylation level and more miRNA target sites.

**Conclusion:**

This is the first genome-wide investigation of the combined regulation of gene expression. Our results supported a complementary regulation between DNA methylation (transcriptional level) and miRNA function (post-transcriptional level) in the human genome. The results were helpful for our understanding of the evolutionary forces towards organisms' complexity beyond traditional sequence level investigation.

## Background

Epigenetics refers to the heritable changes that modify DNA or associated proteins without changing the DNA sequence itself [1]. It has been commonly accepted that both epigenetic mechanisms - DNA methylation modification at the gene's promoter regions (5' of the gene) and microRNA (miRNA) regulation at the 3' untranslated regions (3' UTRs) - are important in gene expression regulation. DNA methylation has been popularly investigated due to its heritable epigenetic modifications of the genome and has been implicated in the regulation of most cellular processes. These include embryonic development, transcription, chromatin structure, X chromosome inactivation, genomic imprinting and chromosome stability [2-6]. Aberrant DNA methylation has been frequently reported to influence gene expression and subsequently cause various human diseases, especially cancer [7-9]. The causal relationship between variation in promoter DNA methylation and difference in gene regulation has been well recognized [10,11]. Recent work [12] revealed that hypermethylation at promoter CpG sites typically results in a lower transcription level of downstream genes. When methylation was experimentally removed from a gene's promoter region, its transcription level would often be higher [13]. Among the ~28 million CpG dinucleotide sites that are susceptible to methylation in the human genome, approximately 10% are in the promoter regions of genes, in which they may physically obstruct the binding of transcriptional proteins to the gene or may be indirectly regulated by the recruitment of methyl-CpG-binding domain proteins through cytosine methylation [14-16]. The repression role in gene expression regulation by methylation modification in a gene's promoter region has been reinforced by current whole genome bisulfite sequencing of the methylomes of more than 20 eukaryotes [17].

miRNAs are a class of small noncoding RNA molecules that regulate eukaryotic gene expression at the post-transcriptional level. They specifically bind mRNAs in their 3' UTRs based on sequence complementation and lead to translational repression and gene silencing [18]. According to release 17 (April 26, 2011) of the miRNA database miRBase [19], there are 16,772 miRNA gene loci in 153 species and 19,724 distinct mature miRNA sequences [20]. Among them, the human genome encodes 1424 miRNA sequences, which may target approximately 60% of human protein-coding genes [21]. This huge number of miRNAs discovered so far indicates that many biological processes, including cell cycle control, cell growth and differentiation, apoptosis, and embryo development, are controlled by miRNA-mediated gene expression regulation [22].

Although there have been many important advances in understanding gene silencing roles at the transcriptional level through DNA methylation modification and at the post-transcriptional level through miRNA regulation, it remains unclear how these two major mechanisms cooperate at the genome-wide level to influence cellular processes. Thus, a combinatory analysis of these two mechanisms is likely to reveal many important insights into a deeper understanding of gene regulation in cells. Considering that (1) DNA methylation acts on a gene's 5' promoter region, and transcription typically depends on demethylation of the promoter region, and (2) miRNAs target on 3' UTR to suppress gene's post-transcriptional activities, we hypothesized that there exists a functional complementation between transcriptional promoter region methylation regulation and post-transcriptional miRNA regulation. If this hypothesis is valid, we would infer that (1) miRNAs preferentially target genes with a low DNA methylation level at the promoter regions; (2) genes that are controlled by more miRNAs tend to have less promoter methylation regulation. We validated our hypothesis by deeply analyzing human methylome data in two cell lines. To the best of our knowledge, this is the first report of the complementary relationship between DNA methylation regulation and miRNA regulation in a eukaryotic genome. Furthermore, we found that cancer genes tended to be silenced by miRNAs and to escape from DNA methylation suppression, thereby supporting our hypothesis.

## Methods

### Gene annotation

Human and mouse gene structure data was retrieved from the Ensembl database (version 54), including the information of Ensembl gene ID, Ensembl transcript ID, transcript start (bp), transcript end (bp), Ensembl protein ID, 3' UTR start, 3' UTR end, chromosome, and strand. We extracted the promoter region and 3' UTR position information from Gene structure data. If there are multiple transcripts for a gene, the transcription start site (TSS) and 3' UTR of the major transcript were used [23]. We retained only those genes without distant alternative TSS (> 200 bp distance from the major TSS) and without ambiguous 3' UTR regions to avoid the potential inaccurate mapping of the gene expression data and gene structures.

### Analysis of DNA methylation data

The single-base resolution DNA methylation data was retrieved from Lister *et al*. (2009) [15], including whole genome bisulfite sequencing data for two human cell lines: H1 human embryonic stem cells and IMR90 fetal lung fibroblasts. The methylation information for each promoter was extracted by mapping the promoter region (in a range of -1000 to +200 bp from the TSS) to the genome-wide methylation data from the H1/IMR90 cell line.

Based on single-base resolution bisulfite sequencing data, we used methylation broadness to measure the DNA methylation level in specific genome regions, which was calculated as the proportion of methylated CpG sites among the total CpG sites in a sequence (we denote it as "mCG/CG" hereafter).

We also used "normalized" CpG content, the observed over expected CpG ratio (CpG_O/E_) in a sequence, to infer the pattern of DNA methylation in the human genome. CpG_O/E _is a robust measure of the level of DNA methylation on an evolutionary time scale due to specific mutational mechanisms of methylated cytosines [23]. Briefly, methylated cytosines are hypermutable due to their vulnerability to spontaneous deamination, which causes a gradual depletion of CpG dinucleotides from methylated regions over evolutionary time. Consequently, genomic regions that are subject to strong germline DNA methylation (hypermethylated) would decrease the extent of CpG dinucleotide content over time and, thus, have lower-than-expected CpG_O/E_. In contrast, regions that undergo weak germline DNA methylation (hypomethylated) maintain high CpG_O/E_. This measure has been successfully used to indirectly measure historical DNA methylation levels. In particular, the pattern of DNA methylation inferred from CpG_O/E _corresponds well to the actual pattern of DNA methylation in such diverse taxa as human and sea squirt. CpG_O/E _was calculated as the frequency of CpG sites divided by the frequency of C and G [24]. The pattern of DNA methylation inferred from CpG_O/E _corresponds well to the actual pattern of DNA methylation in human stem cells (H1 cell line) and fetal lung fibroblasts (IMR90) [14,15]. Since the DNA methylation level of two strands in any given genomics region are highly correlated, here we used the sense strand to represent the DNA methylation level for a given gene promoter region. Similar results were obtained in this study when we used the methylation level of anti-sense (data not shown).

### Compilation of miRNA targets

The miRNAs and their predicted targets were extracted from R package *RmiR.hsa *[25], including miRNA target site prediction results from 6 sources: miRBase, targetScan, miRanda, tarBase, mirTarget2 and PicTar. In this study, we used the target site prediction results from two approaches: mirTaeget2 and PicTar.

### Analysis of human gene expression data

We obtained the expression data of 409 microarray experiments from McVicker and Green (2010) [26], which were collected from 12 studies [12,13,27-36], representing a wide variety of germ and somatic tissues. As these studies used two different platforms (Affymetrix microarrays hgu133plus2 and hgu133A), we only considered the probe sets shared by both arrays. The methods to process the raw intensity data and to assign the probe sets to genes were described in McVicker and Green (2010) [26]. In total, we assigned an expression intensity of 9858 genes in 409 tissues. Among the 409 tissues, 64 containing germ cells were considered as germline tissues, with the exception of germ cell tumors, embryonic stem cells, and immortalized cell lines (see additional file 1).

Because the above data sets are highly redundant in terms of tissue or cell type, we only used Gene Expression Atlas data to estimate the relative expression broadness (EB, number of tissues where a gene is expressed). This data has been widely used to estimate gene expression broadness. The Affymetrix raw data was downloaded from the website of the authors in reference [36]. It comprised 156 human (U133A/GNF1H) microchip experiments in 79 tissues. The expression level detected by each probe set was obtained as the average difference (AD) value computed from MAS 5.0 algorithm (MAS5) [37]. The AD values were averaged among replicates. Using the annotation tables from the original study [36,38] and the Ensembl EnsMart tool, we mapped the probe IDs used in the microarray experiments to Ensemble gene identifiers. In approximately 20% of the cases, multiple probes in the microarray targeted onto a single gene. The expression intensities of multiple probes that corresponded to one gene were averaged after discarding all the low-confidence probe sets (indicated by a suffix of ''_x_at'' or ''_s_at'' in the Affymetrix IDs) [39]. In this study, we used an AD value of 200 as the threshold to calculate the EB, as we did in our previous work [23].

The gene expression data of two human cell lines H1 and IMR90 was obtained from reference [15]. The expression data was generated by a whole RNA sequencing (RNA-Seq) approach. The reads per kilobase of transcript per million reads (RPKM) were used to represent the expression level of each gene.

### Cancer genes

We retrieved 427 human cancer genes and their annotations from the Cancer Gene Census database (CGC, 2010-03-30 version) [40]. Since a cancer gene may act in a dominant or recessive manner [41,42], we classified these 427 cancer genes as two groups, i.e., dominant gene group (337 genes) and recessive gene group (85 genes), according to their annotations in the CGC database. There were 5 genes with ambiguous classification in the database and they were excluded in this analysis.

### Human-specific insertion/deletion (indel) events in 3' UTRs

We identified the human-specific indel events in 3' UTR regions as described in [43]. The 17-way vertebrate alignment, i.e., multiple alignments of 16 vertebrate genomes to the human genome (hg18), was obtained from [44].

An in-house Perl script was used to extract the orthologous 3' UTR alignment information and to identify the human-specific indel events. Human-specific insertion event rate and deletion event rate in the 3' UTR regions were calculated based on percent nucleotide difference. The indel rate equals to the sum of the lengths of all indels in the aligned human sequences divided by the total length of the aligned sequences.

## Results and discussion

### Correlation between gene expression level and promoter DNA methylation

Although methylation of gene's promoter regions has long been considered a suppressor of gene expression [17,45], it still remains unclear to which extent the promoter's DNA methylation contributes to the influence of gene expression level [45,46]. For example, most promoters having CpG islands (CGIs) remain unmethylated even in cells that do not express the corresponding gene. On the contrary, most CpG-poor promoters are hypermethylated even in somatic cells where the genes are expressed [47]. What is equally uncertain is the contribution of promoter methylation to the tissue-specific gene expression. Although many studies have shown the tissue-specific differentially methylated regions (T-DMRs) could connect to the gene expression reprogramming in different tissues or developmental stages, others failed to demonstrate such a connection based on the analysis of a small set of genes [48,49].To better understand the relationship between DNA methylation regulation and the gene expression regulation through miRNA targeting, we explored to what extent promoter methylation affects the gene expression level using the genome-wide data set collected in this study. We used two independent measurements, i.e., methylation broadness and normalized CpG content (CpG_O/E_), to test the correlation of promoter methylation and gene expression level.

**Figure 1:**
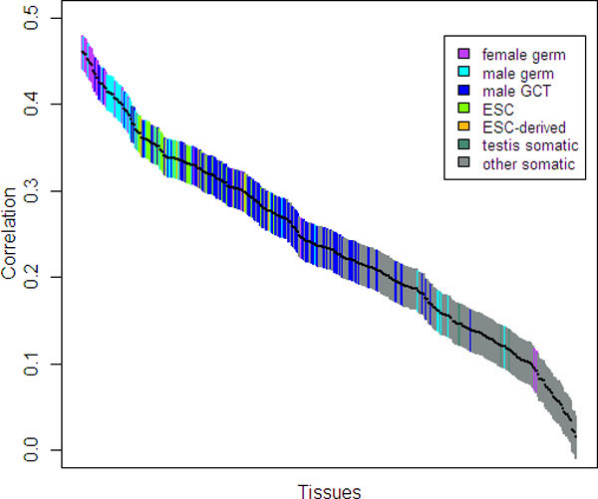
**Pairwise correlations between gene expression and CpG_O/E _ratio in the promoter regions of genes with high tissue differentiation**. Each of the 409 tissue samples is represented by a single bar. Color indicates one of the seven tissue types. GCT: germ cell tumors. ESC: embryonic stem cells. Bars are ordered from left to right by the correlation coefficient value, and their vertical extent indicates the 95% confidence interval.

First, we calculated the broadness of DNA methylation in each gene promoter region in human H1 embryonic stem cells and IMR90 fetal lung fibroblasts, based on the recently published whole genome single-base resolution methylome data [15]. Methylation broadness measures the fraction of cytosine sites detected as methylated in a given DNA segment, which is calculated as the proportion of methylated sites over the total sites in a sequence (termed as mCG/CG) [17]. We calculated the pairwise correlation between promoter DNA methylation and gene expression level. We found gene expression intensity was significantly and negatively correlated with the methylation level in the promoter regions, both in H1 cells (ρ = -0.468, *P <*10^-15^) and in IMR90 cells (ρ = -0.473, *P <*10^-15^). Next, we used CpG_O/E _to approximately infer the pattern of DNA methylation in the human genome. As a robust measurement of the level of germline DNA methylation on an evolutionary time scale [24], low CpG_O/E _and high CpG_O/E _reflect hypermethylation and hypomethylation, respectively. We calculated the correlation between CpG_O/E _and gene expression level for a wide range of tissues. As shown in Figure 1, gene expression in most germline tissues was positively correlated with CpG_O/E_. Remarkably, we found the correlation is more significant in female germline tissues than in male germline tissues. The average gene expression intensity in all germline tissues is also significantly correlated with promoter CpG_O/E _(ρ = 0.37, *P <*10^-15^). Our results also showed either weak correlation or even no significant correlation among most somatic tissues (Figure 1). In summary, using different DNA methylation measurements, we found methylation level in a gene's promoter regions was negatively correlated with expression level at the whole genome level. It is worth noting that we found a more significant correlation between gene promoter DNA methylation level and gene expression level than the previous studies [3,15]. One possible reason is that we only used the genes with unique TSS or largely overlapping promoter regions (see Methods).

### miRNAs preferentially target the genes with low DNA methylation level at the promoter regions

We next tested the hypothesis that a functional complementation exists between transcriptional promoter region methylation regulation and post-transcriptional microRNA regulation. We retrieved unique miRNAs and their target sites for each human gene based on the predicted miRNA binding sites using mirTarget2 [50] and PicTar [51] algorithms. We chose these two algorithms because most of the randomly selected miRNA targets predicted by mirTarget2 and PicTar have been validated as true targets [50,52]. Genes that have long 3' UTRs are likely to be regulated by more miRNAs [53]; thus, we treated the 3' UTR length as a proxy of the number of miRNA target sites for an additional correlation analysis.

**Figure 2:**
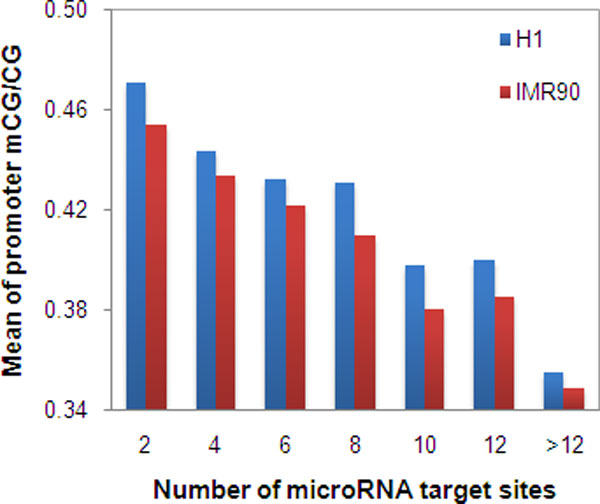
**The correlation between methylation level in promoter regions and number of microRNA target sites**. The number of microRNA target sites in each gene was predicted by mirTarget2. The methylation data was from the methylomes at base resolution of two human cells [15].

**Figure 3:**
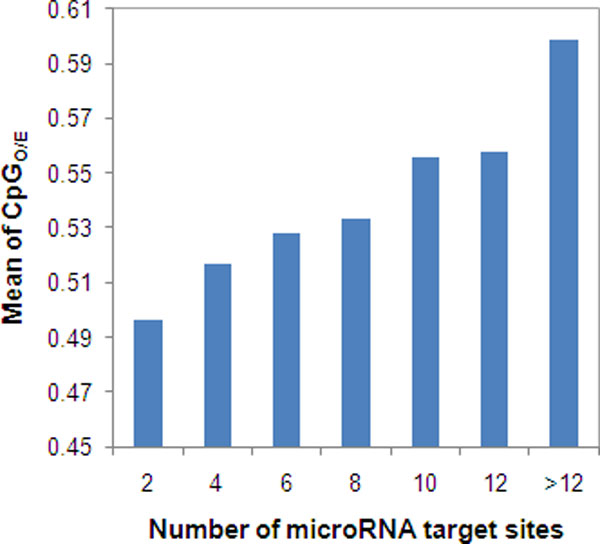
**The correlation between CpG_O/E _ratio in promoter regions and number of microRNA target sites**. The number of miRNA target sites in each gene was predicted by mirTarget2.

There were 12,730 genes that had both miRNA target prediction by mirTarget2 and promoter methylation measured using human H1 cells. Using this dataset, we found a significant negative correlation between gene promoter methylation and number of miRNA target sites (Spearman's ρ = -0.29, *P *< 10^-15^) (Table 1, Figure 2). Similarly, we found a significant negative correlation between gene promoter methylation and number of miRNA target sites (ρ = -0.26, *P *< 10^-15^) based on the 12,731 genes having both miRNA target prediction by mirTarget2 and promoter methylation from methylome of human IMR90 cells (Table 1, Figure 2). Moreover, using the CpG_O/E _value in the promoter regions as a proxy of the promoter methylation level in germline cells, we found a significant positive correlation between CpG_O/E _and the number of miRNA target sites (ρ = 0.29, *P *< 10^-15^) (Table 1, Figure 3). This positive correlation between CpG_O/E _and the number of miRNA target sites is consistent with the negative correlations above, because CpG_O/E _reversely reflects the promoter methylation level. Finally, when we used the miRNA target site data predicted by PicTar, we had very similar results (Table 1), indicating our findings are reliable.

**Table 1:** Spearman's rank correlation coefficients (ρs) and partial correlations between gene's promoter methylation level and the number of microRNA target sites

	**Number of microRNA target sites by mirTarget2 (*Nt*)**		**Number of microRNA target sites by PicTar (*Np*)**		**3' UTR length (*UL*)**	
			
**Promoter methylation level (*m*)**	**Correlation ρ_s _(*m, Nt*)**	**Partial correlation ρ_s _(*m,Nt|gene expression level)***	**Correlation ρ_s _(*m, Np*)**	**Partial correlation ρs (*m,Np|gene expression level)***	**Correlation ρ_s _(*m, UL*)**	**Partial correlation ρ_s _(*m,UL|gene expression level)***
			
Promoter mCG/CG (H1)	-0.29***	-0.19***	-0.29***	-0.22***	-0.31***	-0.22***
Promoter mCG/CG (IMR90)	-0.26***	-0.19***	-0.28***	-0.19***	-0.29***	-0.20***
Promoter CpG_O/E_	0.29***	0.16 ***^†^	0.28***	0.18 ***^†^	0.31***	0.19 ***^†^

**^†^**A germline tissue (FETAL.OVARY.14.4WK) having the most significant correlation between expression level and promoter region CpG**_O/E _**ratio was selected [30].*** ***P ***< 10**^-15^**.

We further used the 3' UTR length to approximately measure the number of miRNA target sites. Consistent with the above results, we found negative correlations between 3' UTR length and promoter methylation level in both human methylomes (H1 and IMR90) (Table 1). This analysis revealed that the genes with a higher promoter methylation level tended to have shorter 3' UTRs at the genome level.

We questioned whether the observed correlations above are unique in the human genome. Thus, we investigated the relationship between promoter DNA methylation level and the number of miRNA target sites in mice. We retrieved the corresponding gene structure data from the ENSEMBL database. The data processes that included the definition of TSS and estimation of 3' UTR length were the same as in humans, as described in the Methods section. We found a highly significant correlation between promoter CpG_O/E _and 3' UTR length (Spearman's ρ = 0.24; *P *< 10^-15^), indicating that the negative correlation pattern between promoter region methylation and number of miRNA target sites still holds in mice. Since mammalian genomes share many CpG island features in their promoter regions [4], it is likely that the observed correlation is common in mammals, or even in many vertebrates.

### Enrichment of miRNA targets among genes with lower promoter methylation level is not a by-product of gene expression level, expression broadness or the number of transcription factor binding sites

We next specifically investigated whether the above observed enrichment of miRNA targets among genes with a lower promoter methylation level was a by-product of ancillary features of the analyzed gene sets. The results from the following analyses indicated this was not the by-product.

First, we asked whether the relationship between DNA methylation and miRNA regulation could be explained by the underlying gene expression levels since the DNA methylation of a gene's promoter regions and gene expression level is correlated in the majority of eukaryotes, and gene expression level is often positively correlated with the number of miRNA target sites. We estimated partial correlations [54] between DNA methylation and number of miRNA target sites after removing the contributions of gene expression level. The corresponding corrections were still highly significant, suggesting that covariance between DNA methylation (or the number of miRNA target sites) and gene expression level could not account for the observed relationships between DNA methylation and the number of miRNA target sites. As shown in Table 1. Although the partial correlations between DNA methylation and miRNA regulation decreased after removing the effects of gene expression level, they still showed high significance

Second, broadly expressed genes tended to avoid miRNA regulation [55,56], implying that the correlation between promoter methylation and miRNA regulation could have been affected by the greater chance of higher DNA methylation level in broadly expressed genes' promoter regions.

**Figure 4:**
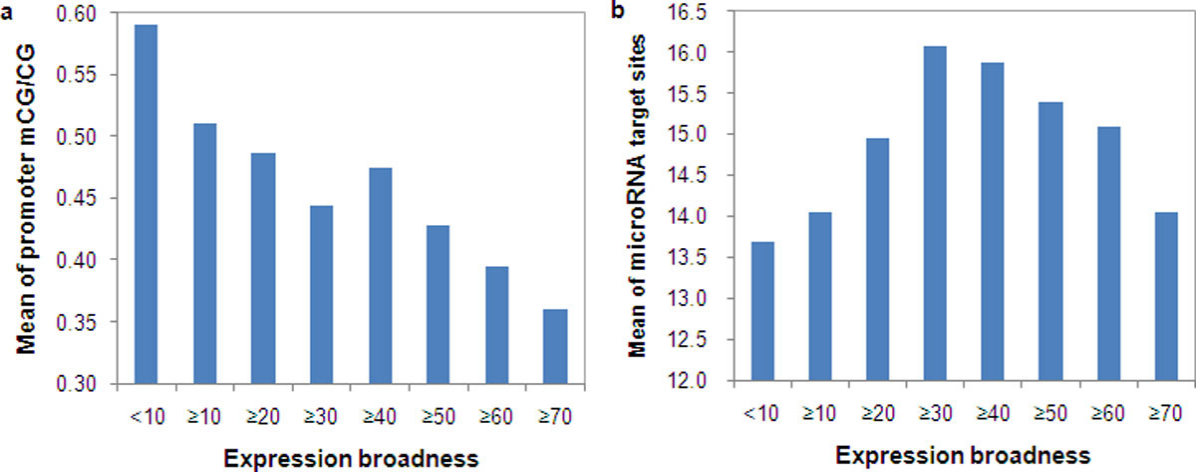
**Distribution of promoter methylation level and number of microRNA target sites in genes by their expression broadness**. (a) Negative correlation between promoter methylation level (mCG/CG) in human cell H1 and expression broadness. (b) microRNAs preferentially target the genes with intermediate expression broadness.

We indeed found the promoter methylation level was negatively correlated with gene expression broadness (EB) (for mCG/CG using H1 methylome data, Spearman's ρ = -0.19, *P *< 10^-15^; for CpG_O/E_, ρ = 0.22, *P *< 10^-15^) (Figure 4a). However, no significant correlation between the number of miRNA target sites and EB was observed (for miRNA target sites based on MirTarget2, ρ = -0.003, *P *= > 0.1) (Figure 4b), and only a very weak correlation between the length of UTRs and EB (ρ = 0.03, *P *= 0.002) was observed. We had similar results using the methylation data of IMR90 and/or using the predicted miRNA target sites by PicTar (data not shown). Therefore, the effect of EB on the correlation of promoter methylation level and miRNA target sites could be largely ruled out.

Third, recent studies found genes with more transcription factor binding sites (TFBS) have a higher probability to be controlled by miRNAs [57].We examined whether the promoter methylation levels are correlated with the number of TFBS. We extracted the TFBS data from [58]. A total of 22,067 genes had both TFBS and promoter methylation data. We found the correlation between TFBS and promoter methylation was very weak (Spearman's ρ = -0.016 for TFBS and CpG_OE_; ρ = -0.07 for TFBS and mCG/CG using H1 mythylome data). This observation suggested that the correlations between promoter methylation level and the number of miRNA targets was not a side effect of the correlation of TFBS site number and the number of miRNA target sites.

Finally, a previous study found that gene evolutionary rates were negatively correlated with the number of their regulatory miRNAs [53]. Therefore, we speculated genes with stronger promoter methylation repression (tend to be regulated by fewer miRNAs) might have evolved faster in their 3' UTRs and could have insertion or deletion bias. A possible mechanism of the negative correlation between promoter methylation and the number of regulatory miRNAs is that genes with hypermethylated promoters may in turn shorten their 3' UTRs to reduce possible miRNA regulation. We tested this hypothesis by the following analyses. We extracted the human-mouse one-to-one orthologous 3' UTR sequences from PACdb [59] and aligned these orthologous sequences using the computer program Clustal W [60]. We calculated the substitution rates per site (termed as *K_3u_*) based on the Kimura's two-parameter model [61]. We found a weak positive correlation between *K_3u _*and the promoter methylation level (Spearman's ρ = 0.15, *P *< 10^-15 ^between *K_3u _*and mCG/CG using H1 mythylome data; ρ = -0.1, *P *< 10^-10 ^between *K_3u _*and CpG_O/E_), indicating promoter hypermethylated genes tended to evolve faster in their 3' UTRs. We identified the human-specific insertion rate and deletion rate for the 3' UTRs of all genes (see Methods). However, there was no evidence to show that promoter hypermethylated genes tended to shorten their 3' UTR length (*P *> 0.1). Further studies of promoter methylation and 3' UTR evolution will be needed to uncover the underlying mechanisms of the connection between promoter methylation level and the number of miRNA target sites.

### miRNA targets are significantly enriched in genes located in differentially methylated regions or partially methylated domains

**Figure 5:**
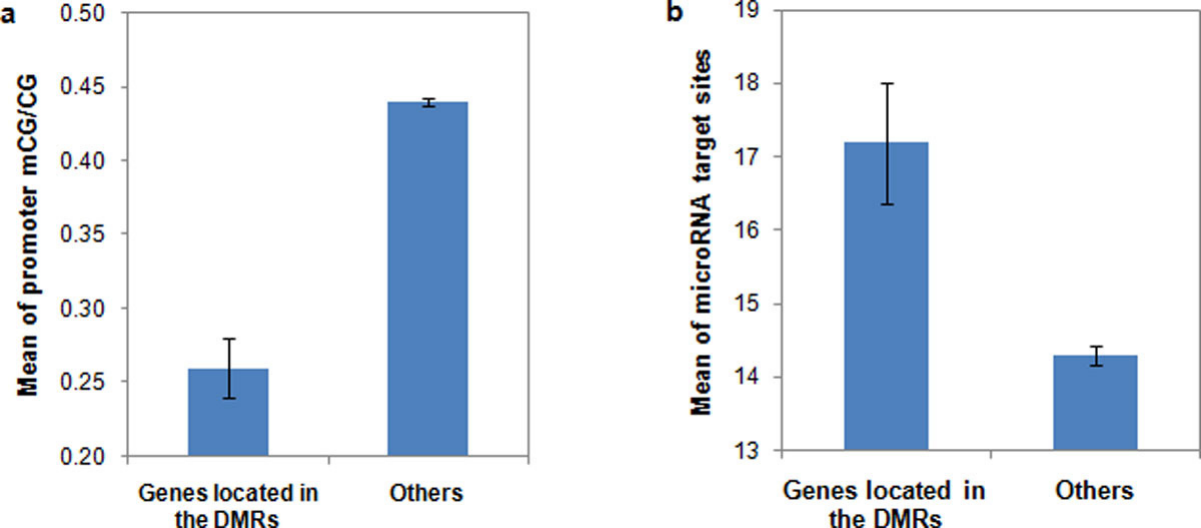
**Genes located in differentially methylated regions (DMRs) tend to have low methylation level measured by promoter mCG/CG and more microRNA target sites**. Error bar: standard error.

Some genes may belong to a specific group of genes that are preferentially regulated by miRNAs or promoter region methylation. It is interesting to investigate the functional complementation between transcriptional promoter methylation and post-transcriptional miRNA regulation in such groups of genes. Specifically, we identified the genes located in differentially methylated regions (DMRs) and partially methylated domains (PMDs) using the data from Lister et al. [15]. According to Lister et al. [15], the DMRs were identified as the regions of the genome enriched for sites of higher levels of DNA methylation in IMR90 relative to H1 by Fisher's Exact Test. There were 491 regions considered as DMRs using the methylome data from H1 and IMR90 cell lines. For the genes located at either DMRs or other genomic regions, we calculated the average number of miRNA target sites and average value of promoter methylation level, respectively. Using the H1 methylome data, on average, genes located at the DMRs and other regions had mCG/CG ratios of 0.26 and 0.44 (*P *< 10^-15^, Mann-Whitney U test) (Figure 5a), and 17.2 and 14.3 miRNA targets sites (*P *< 10^-6^, Mann-Whitney U test) (Figure 5b), respectively. These findings indicate that genes located in DMRs tended to maintain a low methylation level, whereas they might be regulated by more miRNAs. Therefore, there exists a negative correlation between DNA methylation level and the number of miRNA target sites.

**Figure 6:**
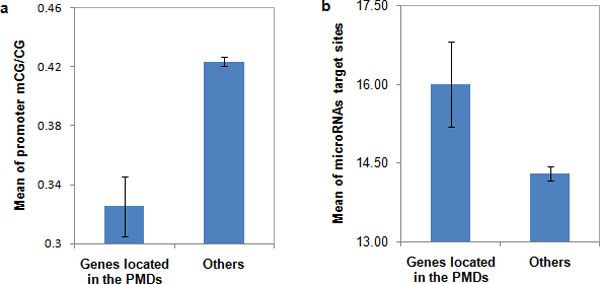
**Genes located in partially methylated domains (PMDs) tend to have low methylation level measured by promoter mCG/CG and more microRNA target sites**. Error bar: standard error.

Lister et al. showed a trend of decreased level of methylation level in PMDs (partially methylated domains in IMR90 cell line, contiguous regions with an average methylation level less than 70%). We calculated the average number of miRNA target sites in PMDs and other genomic regions. As expected, genes located in PMDs had a lower promoter methylation level (*P *< 10^-4^) and were regulated by more miRNAs (*P *< 10^-6^) (Figure 6). This result again demonstrated a negative correlation existed between promoter methylation level and the number of miRNA target sites.

### DNA methylation and miRNA regulation in cancer genes

Cancer is a common complex disease, and many genes have been reported as involved in the development of cancer. Since cancer genes have been extensively studied and often found to be regulated by miRNAs, it is interesting to examine whether the cancer genes are more likely to have low methylation in accordance with our hypothesis and our observations above. To test this hypothesis, we retrieved human cancer genes and their annotations from the CGC database and compared the cancer genes and other genes by their numbers of miRNA target sites, normalized methylation level, CpG_O/E _and number of CGIs in the promoter regions. Table 2 summarizes the results of these analyses. We found that cancer genes tended to have more miRNA target sites than other genes (average 18.60 miRNA target sites for cancer genes versus 14.34 for other genes, *P *< 10^-15^, Mann-Whitney U-test). On the contrary, cancer genes had lower methylation levels than other genes, regardless of whether the methylation level was measured by methylation broadness (mCG/CG), normalized CpG content (CpG_O/E_), or number of CGIs in the promoter regions (Table 2). For example, the normalized methylation level in cancer genes' promoter regions was lower than other genes (average 0.33 for cancer genes versus 0.53 for other genes, *P *< 10^-15^, Mann-Whitney U-test).

**Table 2:** Summary of microRNA target sites and methylation data in gene's promoter regions

Gene categories	Mean of microRNA target sites (gene number)	Mean of mCG/CG (gene number*)	Mean of CpG_O/E _(gene number)	CGI number in promoter region (gene number)
Cancer genes	**18.60 **(379)	**0.33 **(381)	**0.62 **(381)	**0.76 **(412)
Dominant	19.18 (302)	0.33 (299)	0.61 (299)	0.73 (327)
Recessive	16.16 (76)	0.32 (80)	0.66 (80)	**0.87 **(84)
Others	**14.34 **(14,315)	**0.53 **(20,359)	**0.48 **(20,359)	**0.53 **(21,767)

CGI: CpG island. Dominant and Recessive genes are two major cancer gene categories.

*Those genes with CpG reads in their promoter region less than 3 were excluded.

We next compared the features in two major groups of cancer genes: dominant and recessive cancer genes. Among the 427 cancer genes, there were 337 dominant cancer genes and 85 recessive cancer genes based on their annotations in the CGC database. We analyzed their DNA methylation levels and number of miRNA target sites. For a normalized methylation level and CpG_O/E_, no significant difference was detected between the dominant and recessive cancer genes. However, the number of miRNA target sites in the dominant cancer genes (19.18) was larger than that of recessive cancer genes (16.16). Finally, the number of CGIs in the promoter regions of the dominant cancer genes (0.73) was significantly smaller than that of the recessive cancer genes (0.87, *χ*^2 ^test, *P*<10^-15^). These comparisons suggested the different inheritable mechanisms of the dominant and recessive cancer genes in cancer, as we recently examined in the protein-protein interaction level [62].

Collectively, we observed that the promoter region methylation level in cancer genes was negatively correlated with their number of miRNA target sites. This observation still held after filtering the potential confounding effects from gene expression level or expression broadness. This analysis indicated that the cancer genes tended to be silenced by miRNA genes but could escape from DNA methylation suppression.

## Conclusion

To understand how DNA methylation and miRNA regulate the expression of their target genes, many previous exploratory studies have been reported, but all of them focused on the effect of each mechanism on the expression of target genes. In this study, we investigated the relationship between promoter methylation and miRNA regulation at the genome level by taking advantage of recently released human methylome data and high quality miRNA and other gene annotation data. Our results suggested that there is a functional complementation between promoter methylation regulation at the transcription level and miRNA regulation at the post-transcriptional level. Specifically, the genes that are under stronger promoter DNA methylation control tend to avoid miRNA regulation by having fewer miRNA target sites, and vice versa.

From an evolutionary perspective, both recruitment of DNA methylation in a gene's promoter region and the advent of new miRNA genes during the transition from invertebrate to vertebrate contributed to the high complexity of vertebrate organisms and cell types [63-65]. Although many recent studies have greatly improved our understanding of the evolutionary adaptations and conservation of DNA methylation and miRNA regulation, the relationship between DNA methylation and miRNA regulation, and how these two mechanisms dynamically influence each other's evolution and function, remain poorly understood. The results supporting complementary regulation between DNA methylation and miRNA function in this study provided the first attempt to uncover such an important and complex regulation system, which will help us understand the evolutionary forces towards organisms' complexity beyond traditional sequence level investigation.

## Competing interests

The authors declare that they have no competing interests.

## Authors' contributions

ZS designed the study, carried out the data analysis and drafted the manuscript. JX participated in the data analysis and drafted the manuscript. ZZ conceived of the study, designed the project, participated in the data analysis and drafted the manuscript. All authors read and approved the final manuscript.

## Supplementary Material

Additional file 1 **The gene expression intensities in germline tissues**. Totally 6569 genes can be assigned the expression intensities in 64 tissues. CpG_O/E _was calculated for the promoter region (-1000 bp to +200 bp relative to the TSS) of each gene. Click here for file
